# Receptor for Advanced Glycation End Products (RAGE) Serves a Protective Role during *Klebsiella pneumoniae* - Induced Pneumonia

**DOI:** 10.1371/journal.pone.0141000

**Published:** 2016-01-29

**Authors:** Ahmed Achouiti, Alex F. de Vos, Cornelis van ‘t Veer, Sandrine Florquin, Michael W. Tanck, Peter P. Nawroth, Angelika Bierhaus, Tom van der Poll, Marieke A. D. van Zoelen

**Affiliations:** 1 Center for Infection and Immunity Amsterdam (CINIMA), Academic Medical Center, University of Amsterdam, Amsterdam, the Netherlands; 2 Center for Experimental and Molecular Medicine (CEMM), Academic Medical Center, University of Amsterdam, Amsterdam, the Netherlands; 3 Department of Pathology, Academic Medical Center, University of Amsterdam, Amsterdam, the Netherlands; 4 Department of Clinical Epidemiology, Biostatistics and Bioinformatics, Academic Medical Center, University of Amsterdam, Amsterdam, the Netherlands; 5 Department of Internal Medicine and Clinical Chemistry, University of Heidelberg, Heidelberg, Germany; 6 Laboratory of Biomedical Science, Feinstein Institute for Medical Research, North Shore Long Island University Hospital, Manhassat, New York, United States of America; 7 Division of Internal Medicine and Infectious Diseases, University Medical Center of Utrecht, Utrecht, the Netherlands; 8 Laboratory of Translational Immunology (LTI), University Medical Center of Utrecht, Utrecht, the Netherlands; University of North Dakota, UNITED STATES

## Abstract

*Klebsiella* species is the second most commonly isolated gram-negative organism in sepsis and a frequent causative pathogen in pneumonia. The receptor for advanced glycation end products (RAGE) is expressed on different cell types and plays a key role in diverse inflammatory responses. We here aimed to investigate the role of RAGE in the host response to *Klebsiella* (*K*.) *pneumoniae* pneumonia and intransally inoculated *rage* gene deficient (RAGE^-/-^) and normal wild-type (Wt) mice with *K*. *pneumoniae*. *Klebsiella* pneumonia resulted in an increased pulmonary expression of RAGE. Furthermore, the high-affinity RAGE ligand high mobility group box-1 was upregulated during *K*. *pneumoniae* pneumonia. RAGE deficiency impaired host defense as reflected by a worsened survival, increased bacterial outgrowth and dissemination in RAGE^-/-^ mice. RAGE^-/-^ neutrophils showed a diminished phagocytosing capacity of live *K*. *pneumoniae in vitro*. Relative to Wt mice, RAGE^-/-^ mice demonstrated similar lung inflammation, and slightly elevated—if any—cytokine and chemokine levels and unchanged hepatocellular injury. In addition, RAGE^-/-^ mice displayed an unaltered response to intranasally instilled *Klebsiella* lipopolysaccharide (LPS) with respect to pulmonary cell recruitment and local release of cytokines and chemokines. These data suggest that (endogenous) RAGE protects against *K*. *pneumoniae* pneumonia. Also, they demonstrate that RAGE contributes to an effective antibacterial defense during *K*. *pneumoniae* pneumonia, at least partly via its participation in the phagocytic properties of professional granulocytes. Additionally, our results indicate that RAGE is not essential for the induction of a local and systemic inflammatory response to either intact *Klebsiella* or *Klebsiella* LPS.

## Introduction

*Klebsiella* (*K*.) *pneumoniae* is a major cause of pneumonia [1,2] and the second most common cause of Gram-negative sepsis [3,4]. *Klebsiella* infection presents a significant burden on healthcare and is asociated with high morbidity and mortality rates. The increasing microbial resistance of *Klebsiella* species to last-resort antibiotics, resulting in therapy failure and even more higher mortality rates, is an issue of major concern [5]. Therefore it is important to gain more insight into the pathogenesis of *K*. *pneumoniae* pneumonia in order to develop new treatment strategies.

The receptor for advanced glycation end products (RAGE) is a multiligand receptor of the immunoglobulin (Ig) superfamily. RAGE is expressed in all tissues on a wide range of cell types, including cells involved in the innate and adaptive immune system and it plays a key role in diverse inflammatory processes [6,7]. First of all, RAGE can detect endogenous innate danger signals, also named damage-associated molecular patterns (DAMPs) or alarmins, which are structurally diverse proteins rapidly released by the host itself during infection to warn the host for imminent danger [8]. Engagement of these and other ligands to RAGE can induce inflammatory responses via activation of several intracellular signaling cascades, including the nuclear factor-κB pathway [9]. High mobility group box-1 (HMGB1) and S100A12 (or myeloid related protein-6, MRP-6) are known DAMPs that induce an inflammatory response upon binding to RAGE [10–12]. Previously, it was demonstrated that HMGB1 and S100A12 are released in patients with severe sepsis [13–17] and HMGB1 in mice with experimentally induced abdominal sepsis [18]. In addition, RAGE can function as an endothelial adhesion receptor for the leukocyte integrin CD11b/CD18, thereby promoting leukocyte recruitment to the site of infection [19].

In *in vivo* models of abdominal polymicrobial sepsis, RAGE^-/-^ mice demonstrated a diminished lethality after cecal ligation and puncture (CLP) [20,21]. Moreover, anti-RAGE therapy yielded an enhanced survival even when the anti-RAGE antibodies were administered 24 hours after CLP in mice receiving antibiotic treatment [21]. In an abdominal sepsis model that is more suitable to study the influence of an intervention on bacterial outgrowth and dissemination, RAGE contributed to an effective antibacterial defense, thereby limiting the accompanying inflammatory response [22].

Earlier, we found that RAGE deficient (^-/-^) mice are protected against pneumonia caused by the Gram-positive bacterium *Streptococcus* (*S*.) *pneumoniae* as reflected by an enhanced survival, diminished outgrowth at the primary site of infection and a decreased spreading of bacteria to other body compartments together with reduced lung damage [23]. In accordance, Christaki et al. demonstrated that treatment with anti-RAGE antibodies combinated with the antibiotic moxifloxacin protects mice from *S*. *pneumoniae* pneumonia induced mortality [24]. Whereas the Gram-positive *S*. *pneumoniae* is the most commonly isolated pathogen in patients with community-acquired pneumonia, the Gram-negative *K*. *pneumoniae* is a causative organism in both community-acquired and nosocomial pneumonia [25,26]. We here sought to determine the role of RAGE in Gram-negative pneumonia-originating sepsis caused by *K*. *pneumoniae*.

## Materials and Methods

### Animals

10-week-old male RAGE^-/-^ mice were generated as previously described [27] and backcrossed ten times to a C57Bl/6 background. Wild-type C57Bl/6 mice were obtained from Harlan Sprague Dawley Inc. (Horst, The Netherlands). Experimental groups were age- and sex matched and housed in the Animal Research Institute Amsterdam under standard care.

### Ethics statement

This study ware carried out in concordance with the “Wet op de Dierproeven” in the Netherlands. The Institutional Animal Care and Use Committee of the Academic Medical Center, University of Amsterdam, approved all experiments. All efforts were made to minimize suffering. Induction of pneumonia or sterile lung inflammation happened under isoflurane anaesthesia. In the survival study, mice died either as a direct result of the intervention or by humanely euthanization by cervical dislocation when they met human endpoints as reflected by clinical criteria such as signs of excessive weight loss, lethargy and severe unrelieved distress. Clinical signs of distress and mortality were observed two to four times per day during.

### Experimental study design

*K*. *pneumoniae* pneumonia and *Klebsiella* lipopolysaccharide (LPS)-induced lung inflammation were induced as reported previously [28,29]. Wild-type and RAGE^-/-^ mice were inoculated intranasally with 1 x 10^4^ CFUs *K*. *pneumoniae*. Mice were either euthanized at predefined time points or (in survival studies) monitored for 2 weeks. Preparation of lung and liver homogenates, histology, RAGE staining and bronchoalveolar lavage were performed as described before [23,28,29]. Enzyme-linked immunosorbence and cytometric beads multiplex assays, measurements of aspartate aminotransferase (AST) and alanine aminotransferase (ALT) and cell counts were performed as described before [23,28,29].

### Phagocytosis assays

Phagocytosis of *K*. *pneumoniae* was determined as described before [29,30]. In brief, a concentrated *K*. *pneumoniae* preparation was treated for 60 min at 37°C with 50 μg/mL mitomycine-C (Sigma-Aldrich, Zwijndrecht, the Netherlands) to prepare growth-arrested, but alive bacteria. Next, 50 μL of heparinized whole blood from wild-type and RAGE^-/-^ mice was incubated at 37°C (*n* = 8 mice per group) or 4°C (*n* = 4 mice per group) with Alexa647-succinimidyl-ester (Alexa647-SE, Invitrogen, Breda, the Netherlands) labeled, growth-arrested bacteria (end concentration of 1 × 10^7^ CFUs/mL) for 60 min. Phagocytosis was stopped by placing cells on ice and erythrocytes were lysed with ice-cold isotonic NH_4_Cl solution (155 mM NH_4_Cl, 10 mM KHCO_3_, 100 mM EDTA, pH 7.4). Neutrophils were labeled using anti-Gr-1-PE (BD Pharmingen, San Diego, CA). Cells were then washed with FACS-buffer (0.5% BSA, 0.01% NaN3, 0.35 mM EDTA in PBS) after which the degree of phagocytosis of neutrophils was determined using FACSCalibur (Becton Dickinson Immunocytometry, San Jose, CA). Phagocytosis index of each sample was calculated as follows: mean fluorescense of positive cells × % positive cells.

### HMGB1 measurement

For Western blotting of HMGB1, bronchoalveolar lavage fluid (BALF) samples were diluted with 2x Laemmli buffer. After heating, samples were run on 15% polyacrylamide SDS gels and subsequently transferred to blotting membrane (Immobilon P (Pharmacia, Piscataway, NJ) polyvinylidene difluoride membranes). Following blocking with 5% nonfat dry milk proteins (Protifar from Nutricia, Zoetermeer, the Netherlands) in 0.1% Tween 20 phosphate buffered saline (PBS-T), membranes were washed and incubated overnight in primary antibody (rabbit anti-HMGB1 polyclonal antibody) (1 μg/mL, catalog no ab18256, Abcam, Cambridge, United Kingdom) in 1% nonfat dry milk proteins in PBS-T at 4°C. After washing with PBS-T, membranes were probed with peroxidase-labeled secondary antibodies (Cell Signaling Technology, Danvers, MA) for 1 h at room temperature in 1% bovine serum albumin in PBS-T. After washing with PBS-T, membranes were incubated with Lumi-Light^Plus^ Western Blotting Substrate (Roche, Mijdrecht, The Netherlands) and positive bands were detected using a LAS3000 Luminescent image Analyzer dark box (Fujifilm, Tokyo, Japan).

### Statistical analysis

Differences between groups were analyzed by Mann-Whitney *U* test. Data in the figures are expressed as box-and-whisker diagrams depicting the smallest observation, lower quartile, median, upper quartile and largest observation unless indicated otherwise. For survival analysis, Kaplan-Meier analysis was performed followed by a log-rank test. *P* values of *<* 0.05 were considered to represent a statistically significant difference.

## Results

### RAGE expression in the lungs

**Fig 1:**
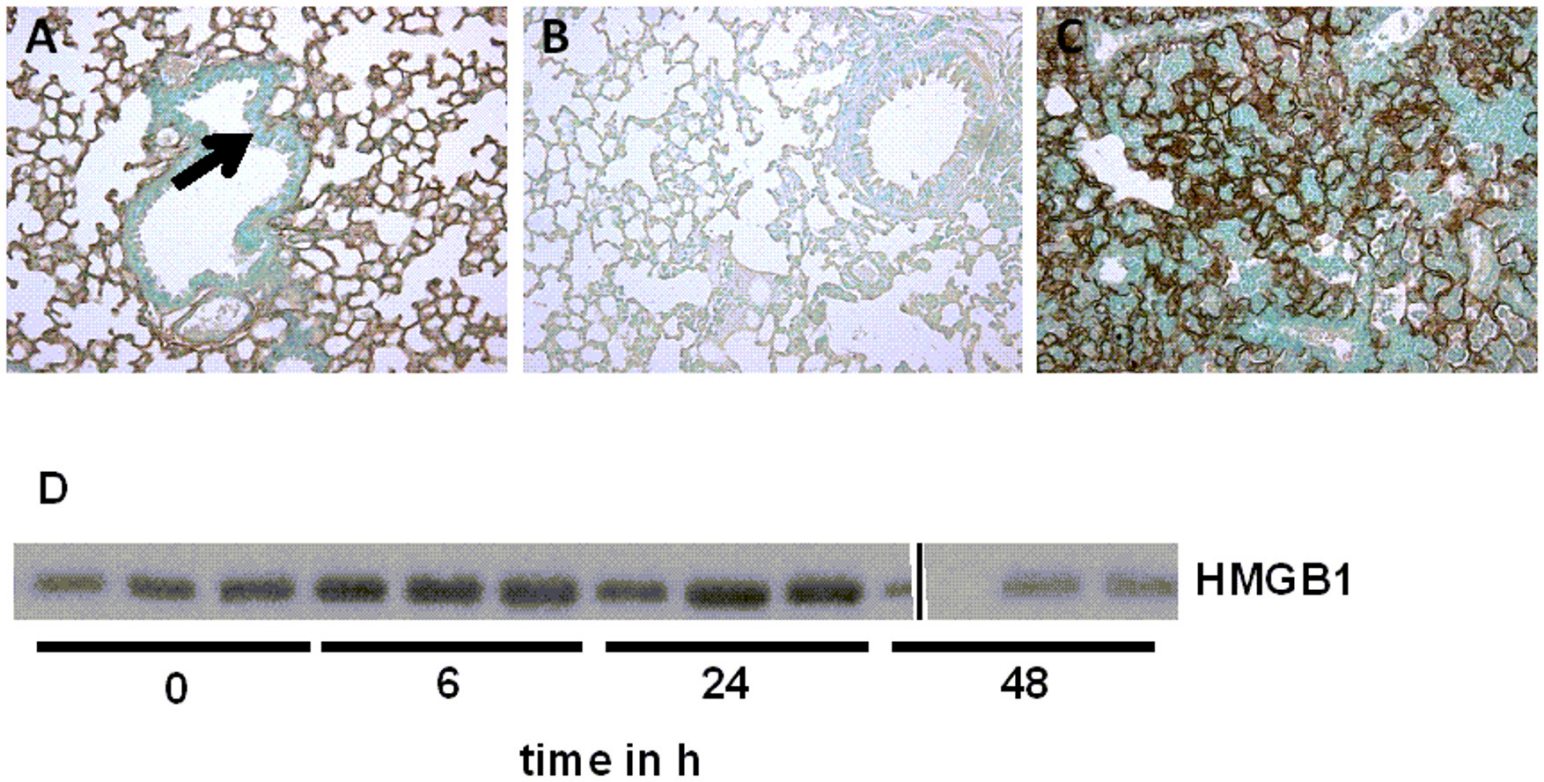
Pulmonary expression of receptor for advanced glycation end products and of its ligand high mobility group box-1 (HMGB1) during *Klebsiella pneumoniae* pneumonia. Representative view of a lung from a normal, uninfected wild-type mouse (A) displaying ubiquitous expression of RAGE on the surface of endothelium. *Arrow* indicates bronchial epithelium, being negative for RAGE staining. B, Absence of RAGE positivity in the lung of a RAGE^-/-^ mouse. C, Lungs from a wild-type mouse 48 h after the inoculation of *K*. *pneumoniae*. Original magnification, x10. D, Western blot was performed for HMGB1 in brochoalveolar lavage fluid (BALF) from wild-type mice at 0, 6, 24 and 48 h after *K*. *pneumoniae* intranasal inoculation (*n* = 3 mice per time point).

To obtain insight into constitutive and *K*. *pneumoniae*-induced RAGE expression, we performed immunohistochemical stainings of RAGE of lung tissue from healthy, uninfected wild-type mice and from wild-type mice after inoculation with *K*. *pneumoniae*. In accordance with the literature [31–34], normal healthy mice showed extensive RAGE staining in their lungs (Fig 1A), being mainly present in the interalveolar septae in an endothelial pattern, while bronchial epithelial cells were negative for RAGE staining (Fig 1A, *arrow*). The specificity of the RAGE staining was confirmed by immunohistochemical analysis of lungs obtained from RAGE^-/-^ mice, used as negative controls (Fig 1B). Lungs from *K*. *pneumoniae* infected mice displayed the same pattern of RAGE positivity as lungs from healthy wild-type mice, *i*.*e*. the interalveolar septae stained positive for RAGE staining with an endothelial pattern; however, RAGE expression was enhanced following pulmonary infection with *K*. *pneumoniae* as reflected by more intense staining (Fig 1C).

### HMGB1 is increased during *K*. *pneumoniae* pneumonia

After having shown that RAGE expression is enhanced during *Klebsiella* pneumonia, we next investigated whether. *K*. *pneumoniae* pneumonia is associated with release of its high-affinity ligand HMGB1 [35]. We previously demonstrated increased HMGB1 concentrations in BALF from the infected site of patients with community-acquired pneumonia [16], in BALF of patients with ventilator-associated pneumonia [36] and in BALF from mice intranasally infected with influenza A virus [37]. Relative to t = 0 h, mice with pneumonia induced by *K*. *pneumoniae* had elevated HMGB1 levels in BALF at 6, 24 and 48 h (Fig 1D).

### RAGE deficiency enhances lethality due to *K*. *pneumoniae* pneumonia

**Fig 2:**
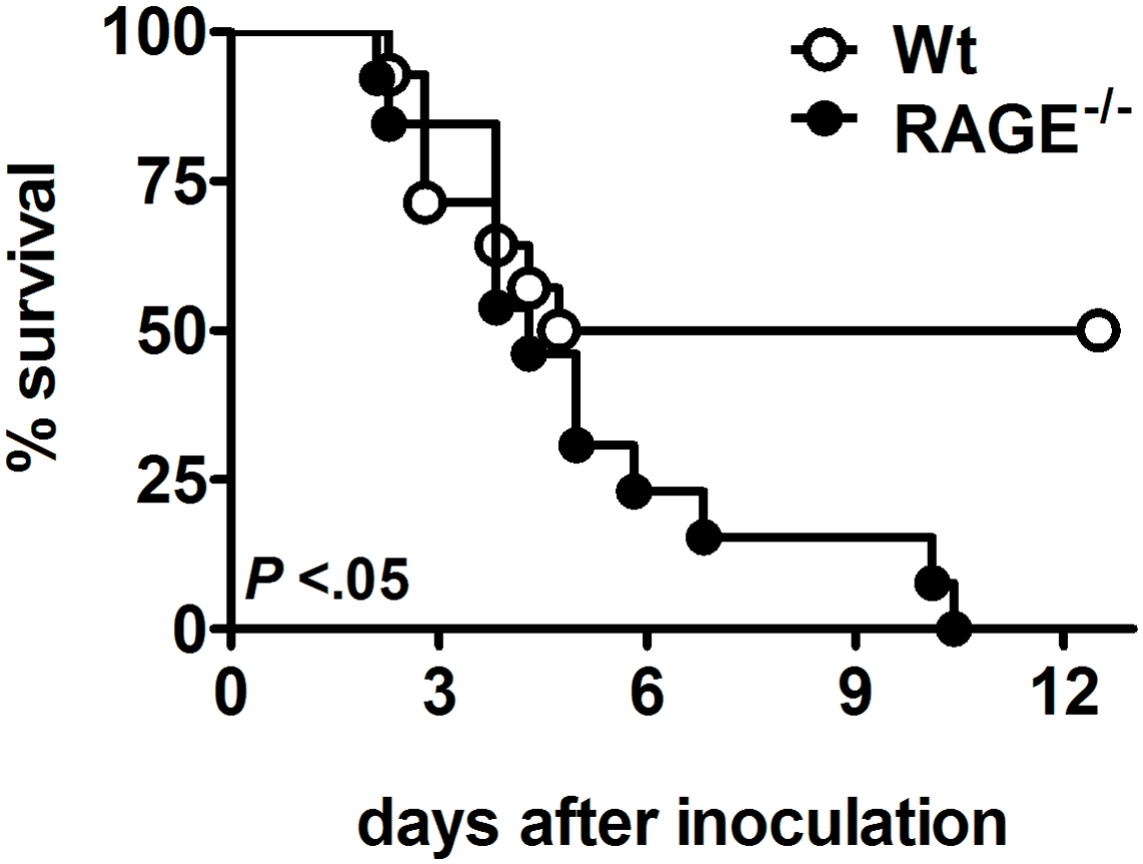
Increased mortality of receptor for advanced glycation end products deficient (RAGE^-/-^) mice during *Klebsiella pneumoniae* pneumonia. Survival of wild-type and RAGE^-/-^ mice after intranasal inoculation with 1 x 10^4^ CFUs *K*. *pneumoniae*. Mortality was assessed for 14 days (*n* = 13–14 mice per genotype).

To study the contribution of RAGE to the outcome of *Klebsiella* pneumonia, wild-type and RAGE^-/-^ mice were intranasally inoculated with *K*. *pneumoniae* and observed for 14 days (Fig 2). While the first deaths occurred after 2 days in both strains, all RAGE^-/-^ mice had died after 10 days, while only 50% of the wild-type mice had died at the end of the observation period (p < 0.05). Thus, RAGE deficiency rendered mice more susceptible to *K*. *pneumoniae* induced death.

### RAGE deficiency facilitates early bacterial outgrowth and dissemination

**Fig 3:**
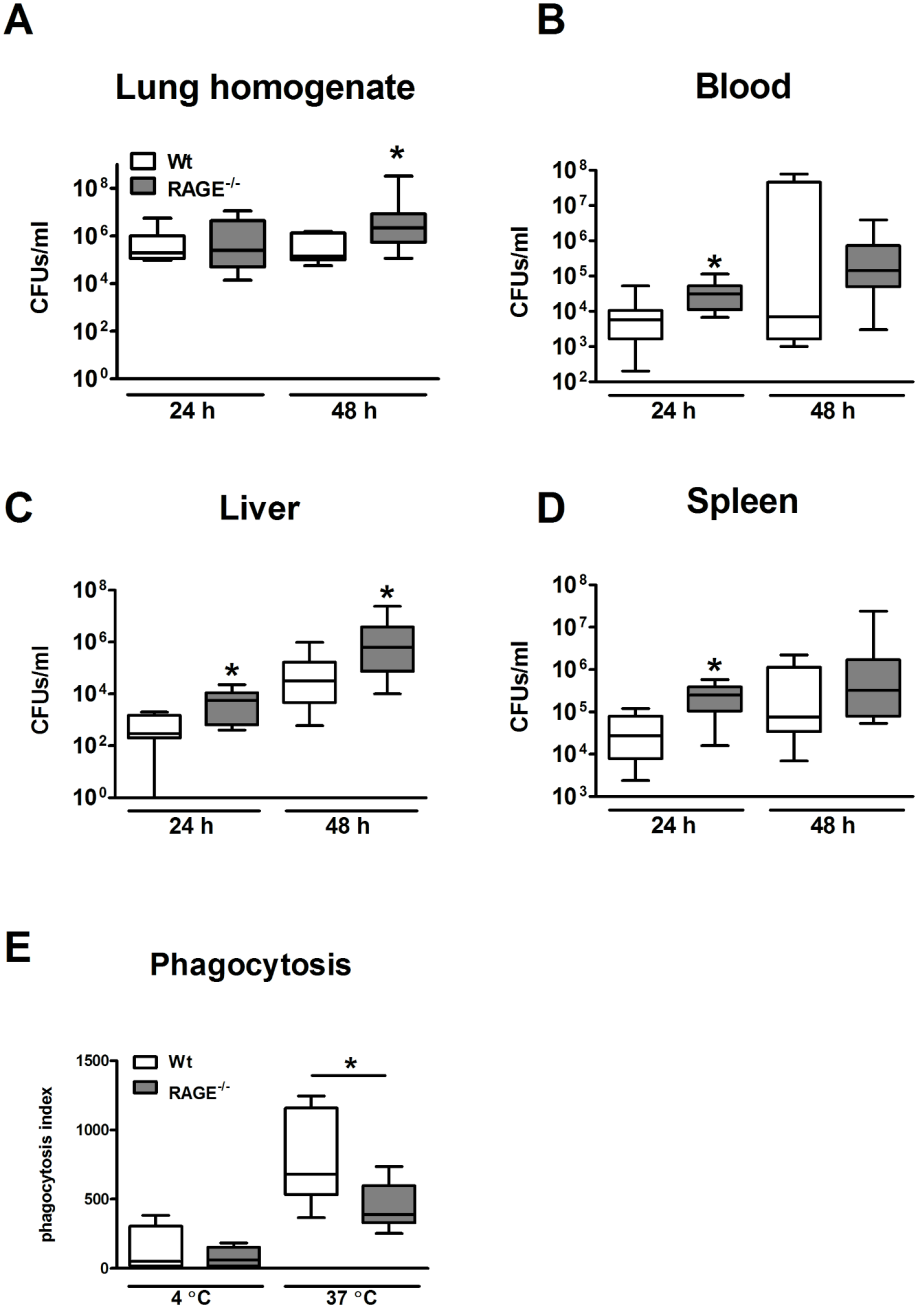
Receptor for advanced glycation end products deficiency enhances local bacterial outgrowth and dissemination during *Klebsiella pneumoniae* pneumonia *in vivo* and reduces *in vitro* phagocytosis of *Klebsiella pneumoniae* by neutrophils. Bacterial loads in lung homogenate (A), blood (B), liver (C) and spleen (D) were determined in wild-type and RAGE^-/-^ mice 24 and 48 h after intranasal inoculation 1 x 10^4^ CFUs *K*. *pneumoniae*. Data are expressed as box-and-whisker diagrams depicting the smallest observation, lower quartile, median, upper quartile and largest observation of 8–10 mice per genotype at each time point. E, Phagocytosis of growth-arrested viable Alexa647-SE labeled *Klebsiella pneumoniae* of neutrophils from wild type and RAGE^-/-^ mice at 37°C or 4°C. Phagocytosis was quantified as described in the Methods section. Data are expressed as box-and-whisker diagrams depicting the smallest observation, lower quartile, median, upper quartile and largest observation (of 4 mice for 4°C and 8 mice 37°C) per genotype. * p < 0.05, compared with wild-type mice.

To obtain insight in the mechanism underlying the higher mortaility of the RAGE^-/-^ mice, we repeated this experiment and sacrificed mice 24 and 48 h after infection (*i*.*e*. directly before the first mice started dying) to enumerate bacterial counts in lungs, blood, liver and spleen. At 24 h after infection, bacterial outgrowth in the lungs were similar in wild-type and RAGE^-/-^ mice. However, after 48 h, the number of *Klebsiella* CFUs was higher in the lungs of RAGE^-/-^ mice when compared to the wild-type mice (Fig 3A; p < 0.05). At 24 h, significantly increased CFU counts were recovered from blood, liver and spleen harvested from the RAGE^-/-^ mice compared to wild-type mice. Liver homogenates from RAGE^-/-^ mice showed increased bacterial loads at 48 h as well (Fig 3B–3D; all p < 0.05). Thus, RAGE limits the outgrowth of *K*. *pneumoniae* in the lungs and the dissemination to the blood stream and distant organs.

### RAGE deficiency results in a decreased phagocytosis of *K*. *pneumoniae* by neutrophils

The increased bacterial load in RAGE^-/-^ mice could be caused by an intrinsic defect of RAGE^-/-^ cells to phagocytose *K*. *pneumoniae*. To investigate this possibility we harvested whole blood from uninfected wild-type and RAGE^-/-^ mice and compared the capacity of neutrophils to phagocytose Alexa647-succinimidyl-ester labeled, but growth-arrested viable *K*. *pneumoniae*. RAGE^-/-^ neutrophils displayed a decreased ability to phagocytose *K*. *pneumoniae* (Fig 3E).

### RAGE deficiency does not impact on lung inflammation during *K*. *pneumoniae* pneumonia

**Fig 4:**
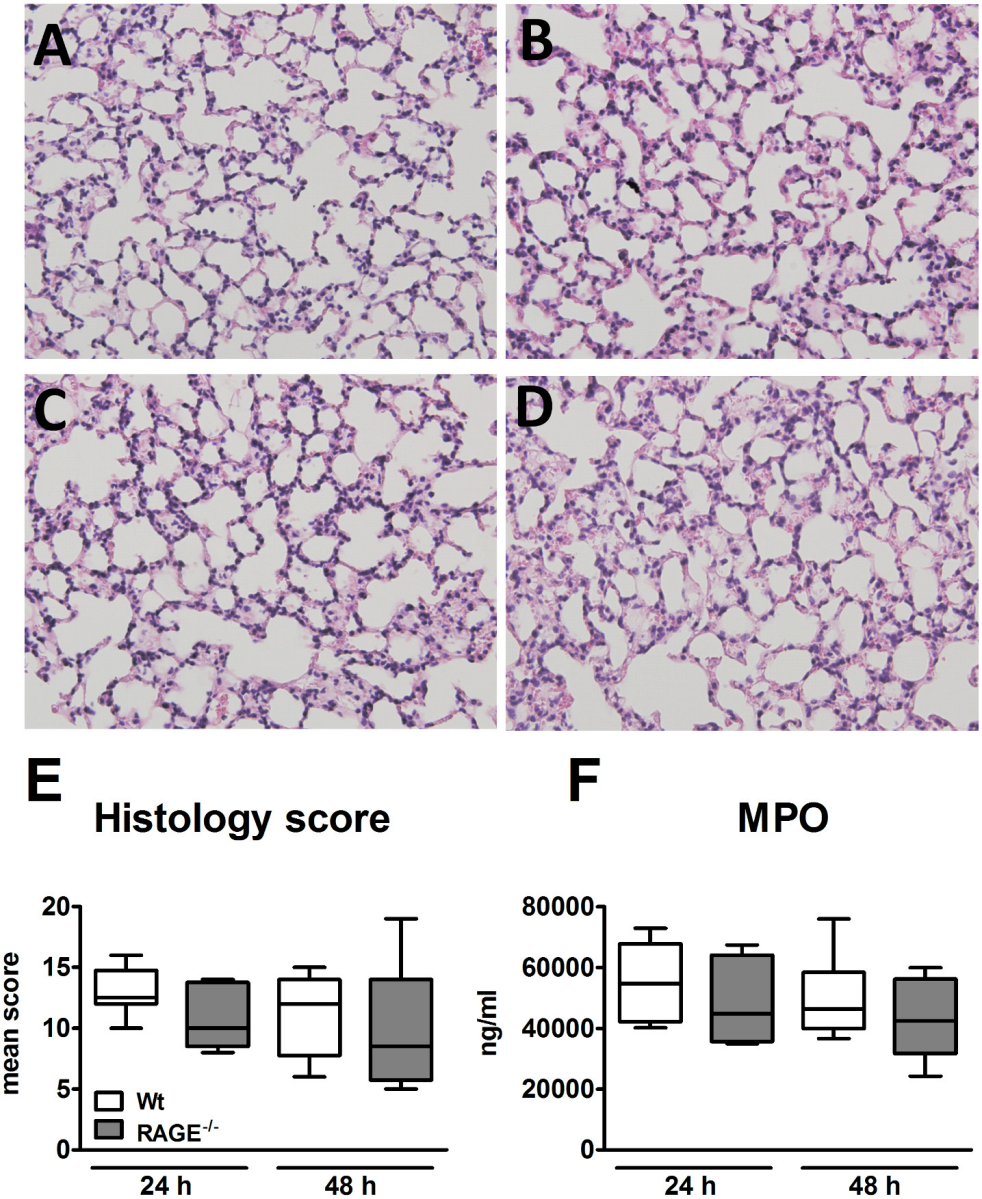
Unchanged lung inflammation during *Klebsiella* pneumonia. Wild-type and RAGE^-/-^ mice were inoculated intranasally with 1 x 10^4^ CFUs *K*. *pneumoniae*. Representative hematoxylin-eosin stainings of lung tissue at 24 (A and B) and 48 (C and D) h post inoculation in wild-type (A and C) and RAGE^-/-^ (B and D) mice. Original magnification, x20. E, Graphical representation of the degree of lung inflammation at 24 and 48 h. F, Myeloperoxidase (MPO) levels in lung tissues. Data are expressed as box-and-whisker diagrams depicting the smallest observation, lower quartile, median, upper quartile and largest observation of 8–10 mice per genotype at each time point. * p < 0.05, compared with wild-type mice.

Considering that RAGE signaling results in sustained cellular activation we were interested to study the role of RAGE in lung inflammation during *Klebsiella* pneumonia. Thus, we analyzed lung tissue slides obtained from wild-type and RAGE^-/-^ mice 24 and 48 h after infection. At both time points, both mouse strains displayed interstitial inflammation together with vasculitis, peri-bronchitis, edema and pleuritis (Fig 4A–4D). Importantly, in contrast to our expectation, the extent of lung inflammation, as determined by the semi-quantitative scoring system, analyzing the severity of vasculitis, bronchitis, edema and pleuritis, was not different between wild-type and RAGE^-/-^ mice (Fig 4E). In addition, myeloperoxidase (MPO) concentrations in lung homogenates of both mouse strains were similar at both time points (Fig 4F), indicating that RAGE deficiency did not influence neutrophil recruitment. Together these data suggest that RAGE does not play a significant role in the lung inflammation that accompanies *Klebsiella* pneumonia.

### Cytokine and chemokine levels

In pulmonary infection, cytokine and chemokine production is an important factor in the host immune response [38,39]. We determined the influence of RAGE deficiency on pulmonary and systemic cytokine and chemokine concentrations during *Klebsiella* pneumonia. Levels of the cytokines TNF-α, IL-6, MCP-1 and IL-10 and of chemokines KC and MIP-2 did not differ between the two mouse strains at 24 h. At 48 h, MCP-1 and KC concentrations were increased in the lungs of the RAGE^-/-^ mice (Table 1; p < 0.05). In plasma, TNF-α, IL-6 and MCP-1 levels were similar between the two genotypes at both time points, while IL-10 was elevated in the RAGE^-/-^ mice at 48 h (Table 1; p < 0.05).

**Table 1:** Cytokine and chemokine levels in lung homogenate and plasma 24 and 48 h after intanasal administration of *Klebsiella pneumoniae*.

Cytokine levels, mean pg/mL ± SE				
	24 h		48 h	
	wild-type mice	RAGE^-/-^ mice	wild-type mice	RAGE^-/-^ mice
	**Lung homogenate (pg/mL)**			
TNF-α	724 ± 187	446 ± 66	1,679 ± 932	5,151 ± 1617
IL-6	1348 ± 252	723 ± 240	706 ± 301	844 ± 223
MCP-1	6172 ± 381	6006 ± 444	2,861 ± 282	4,838 ± 623^b^
IL-10	545 ± 99	482 ± 50	26 ± 3	32 ± 6
KC	13991 ± 1847	13026 ± 1851	11,257 ± 2,251	20,478 ± 3,372^a^
MIP-2	8082 ± 1818	11298 ± 2300	12,085 ± 4,367	7,548 ± 1,467
	**Plasma (pg/mL)**			
TNF-α	87 ± 30	141 ±47	62 ± 18	56 ± 26
IL-6	211 ± 49	211 ± 58	277 ± 159	478 ± 157
MCP-1	827 ± 188	1,777 ± 444	519 ± 139	526 ± 231
IL-10	14 ± 3	21 ± 3	1 ± 1	8 ± 4^a^

Data are means ± SE of 8–10 mice/group at 24 or 48 h after intranasal instillation of 1 x 10^4^ CFU *K*. *pneumoniae*. SE, standard error; RAGE^-/-^, receptor for advanced glycation end products gene deficient; TNF, tumor necrosis factor; IL, interleukin; MCP, monocyte chemoattractant protein-1; KC, keratinocyte-derived chemokine; MIP, macrophage inflammatory protein.

^a^ P < .05, compared with wild-type mice.

^b^ P < .01, compared with wild-type mice.

### Wild-type and RAGE^-/-^ mice display similar hepatocellular injury

**Fig 5:**
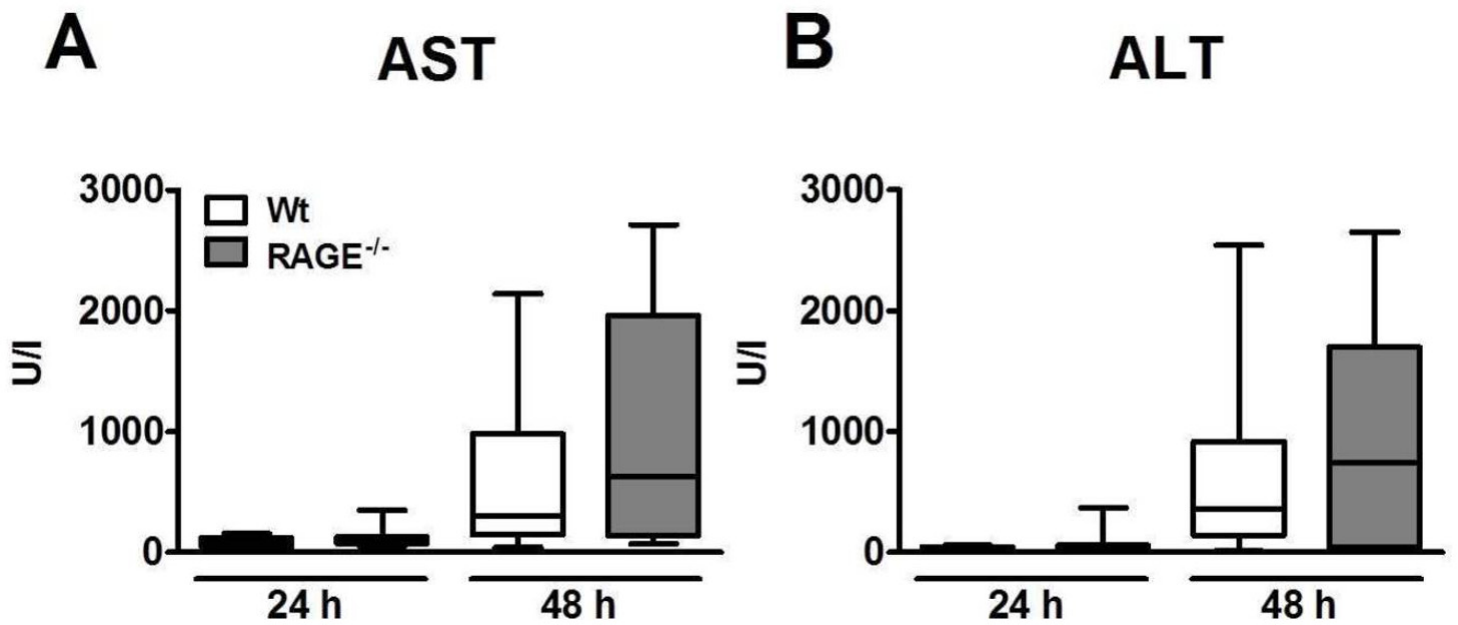
Hepatocellular injury during *Klebsiella pneumoniae* pneumonia. Wild-type and RAGE^-/-^ mice were inoculated intranasally with 1 x 10^4^ CFUs *K*. *pneumoniae* and sacrificed after 24 and 48 h. Aspartate aminotransferase (AST, A) and alanine aminotransferase (ALT, B) in plasma of wild-type and RAGE^-/-^ mice. Data are expressed as box-and-whisker diagrams depicting the smallest observation, lower quartile, median, upper quartile and largest observation of 8–10 mice per genotype at each time point.

This model of *Klebsiella* pneumonia is associated with hepatocellular injury [28]. Considering the enhanced lethality and sustained elevated bacterial loads in liver homogenates in RAGE^-/-^ mice, we were interested to examine the extent of hepatocellular injury in both mouse strains (Fig 5). At 24 h after infection, neither RAGE^-/-^ nor wild-type mice demonstrated elevated plasma concentrations of AST or ALT. At 48 h post infection, both mouse strains had strongly elevated plasma transaminase levels; although these levels tended to be higher in RAGE^-/-^ mice, the differences with wild-type mice did not reach statistical significance.

### RAGE^-/-^ mice demonstrate an unchanged inflammatory response to *K*. *pneumoniae* LPS

In light of the strong expression of RAGE in the lung and its reported role as a receptor mediating proinflammatory effects, we were surprised to find unaltered lung inflammation and (if anything) higher cytokine levels in RAGE^-/-^ mice during *Klebsiella* pneumonia. To obtain further evidence for a modest role of RAGE in the induction of lung inflammation in response to a Gram-negative bacterium, we compared the inflammatory response to *Klebsiella* LPS, administered via the airways, in wild-type and RAGE^-/-^ mice. In these studies we harvested BALF 6 h after *Klebsiella* LPS administration considering that this time point is representative for examining LPS responses in the pulmonary compartment [40–42]. In line with the observations during respiratory tract infection with live *K*. *pneumoniae* in the current study, RAGE^-/-^ mice displayed an unaltered response to intranasally instilled *Klebsiella* LPS with respect to pulmonary cell recruitment and local release of cytokines and chemokines (Table 2).

**Table 2:** Cell counts, cytokine and chemokine levels in bronchoalveolar lavalge fluid after *Klebsiella* lipolysaccharide inoculation.

	wild-type mice	RAGE^-/-^ mice
Cells (x 10^5^/mL ± SE)		
Total cells	5.8 ± 1.0	8.2 ± 1.4
Neutrophils	5.5 ± 1.0	7.7 ± 1.3
Cytokines (pg/mL ± SE)		
TNF-α	5561 ± 819	6212 ± 568
IL-6	2542 ± 973	3989 ± 583
KC	1327 ± 209	870 ± 81
MIP-2	661 ± 79	516 ± 34

Data are means ± SE of 8–10 mice/group at 6 h after intranasal inoculation of 100 μg lipopolysaccharide from *Klebsiella pneumoniae*. RAGE^-/-^, receptor for advanced glycation end products deficient; SE, standard error; TNF, tumor necrosis factor; IL, interleukin; KC, keratinocyte-derived protein; MIP, macrophage inflammatory protein.

## Discussion

*Klebsiella* species is a frequently isolated Gram-negative bacterium in nosocomial as well as in community-acquired pneumonia and in sepsis [1–4] and is associated with high morbidity and mortality. RAGE has the ability to activate signaling pathways, resulting in proinflammatory gene expression upon interaction with several distinct endogenous proinflammatory ligands (DAMPs) [6,7]. Therefore, RAGE may function as a sensor of danger signals leading to a certain amount of inflammation and hence play a beneficial role in bacterial eradication during infection. However, interaction of RAGE with its ligands and the subsequently induced inflammation can also worsen tissue damage, thereby exerting detrimental effects. Our key finding was that RAGE contributes to an effective antibacterial host response during *K*. *pneumoniae* infection. Indeed, RAGE deficiency caused an enhanced outgrowth of *K*. *pneumoniae* at the primary site of infection together with increased spreading of bacteria to other body compartments and an increased mortality. This reduced resistance against *K*. *pneumoniae* in RAGE^-/-^ mice could at least in part be explained by a reduced phagocytosis capacity of RAGE^-/-^ neutrophils.

Theoretically, there is a possibility that underlying mechanisms of the increased mortality in the RAGE^-/-^ mice were not overt at 24 or 48 hours yet. However, we expect this chance to be small, since the first mice died just a few hours after the 48 hour time point. Therefore, we chose to use 24 and 48 hours as time points to investigate possible underlying mechanisms and not after 48 hours to prevent selection of the surviving mice.

In our *K*. *pneumoniae* pneumonia model, RAGE^-/-^ mice had higher levels of the chemokines KC and MCP-1 in their lung homogenates and increased circulating IL-10 concentrations compared to wild-type mice 48 hours after infection, while intranasal inoculation with LPS from *K*. *pneumoniae* did not lead to higher broncho-alveolar chemokine levels in the RAGE^-/-^ mice. The increased chemokine and IL-10 concentrations in the RAGE^-/-^ mice in the *K*. *pneumoniae* pneumonia model could in part be due to the increased bacterial load in the RAGE^-/-^ mice.

Our data on the expression of RAGE in the lungs extend earlier reports in finding broad RAGE expression in normal, healthy lungs [31–34] and an upregulation of pulmonary RAGE expression during interstitial and postobstructive pneumonia [23,32]. The present data are in accordance with our recent reports on enhanced RAGE expression in the lungs of mice infected with *S*. *pneumoniae* [23] or influenza A [37]. In addition, we showed that levels of the RAGE ligand HMGB1 are elevated in BALF during *K*. *pneumoniae* pneumonia, similar to data from mice with intratracheally administered *E*. *coli* induced lung injury [43]. Furthermore, the RAGE ligand S100A12 is released in patients with sepsis [17] and enhanced S100A12 concentrations have been shown in BALF from patients with acute lung injury and from healthy volunteers after LPS inhalation [34], but evidence that a functional *s100a12* gene is not present in the murine genome [44] implies that RAGE-S100A12 ligation does not attribute to the host response to pneumonia in mice.

The current data should be considered in the context of several other studies on the role of RAGE during bacterial infections. Ramsgaard et al. reported that RAGE deficiency leads to a reduced inflammation after intratracheal administration of *E*. *coli* [43]. Compared to wild-type mice, RAGE^-/-^ mice had lower concentrations of neutrophils, protein, MPO, cytokines and chemokines in their BALF. In that study, bacterial outgrowth, dissemination and survival were not reported; therefore the possible role of RAGE in antibacterial defense cannot be determined from this investigation. In a model of polymicrobial abdominal sepsis induced by CLP, RAGE^-/-^ mice had an improved survival together with a reduced NF-κB activation in the peritoneum [20,21]. Moreover, anti-RAGE therapy conferred a survival advantage even when administered 24 h after CLP in mice receiving antibiotic treatment [21]. In the latter study, RAGE deficiency or anti-RAGE therapy was reported not to influence bacterial loads in peritoneal lavage fluid, liver or spleen. It should be noted, however, that in this study, all mice were treated with broad spectrum antibiotics and bacterial loads were only determined in mice that survived (*i*.*e*. not at predefined time points after CLP induction). We previously investigated the role of RAGE during abdominal sepsis induced by *E*. *coli*, showing that RAGE deficiency was associated with an enhanced outgrowth of *E*. *coli* locally and in distant organs together with more severe liver injury [22]. In contrast, in a model of Gram-positive pneumonia caused by *S*. *pneumoniae*, RAGE deficiency was associated with a reduced bacterial outgrowth and dissemination and less severe lung damage [23]. Interestingly, Christaki et al demonstrated that treatment with anti-RAGE antibodies combined with the antibiotic moxifloxacin protects mice from *S*. *pneumoniae* pneumonia induced mortality [24].

One possible explanation for the intriguing observation that RAGE involvement during (Gram-positive) pneumococcal and (Gram-negative) *Klebsiella* pneumonia has opposite effects on bacterial clearance and mortality is that RAGE mediated effects on first-line defense mechanisms may depend on the pathogen. *In vitro* phagocytosis of *Klebsiella* bacteria was decreased in the RAGE^-/-^ neutrophils, which could at least partly account for the higher bacterial loads in the RAGE^-/-^ mice. Of considerable interest, a study identified RAGE as a receptor for LPS derived from various Gram-negative bacteria, including *K*. *pneumoniae* [45]. These authors reported reduced LPS responsiveness of RAGE^-/-^ peritoneal macrophages *in vitro* and attenuated cytokine release and mortality in RAGE^-/-^ mice after intraperitoneal LPS injection *in vivo* [45]. We also previously demonstrated attenuated systemic TNF-α release upon intraperitoneal LPS administration to RAGE^-/-^ mice when compared to wild-type mice [22]. We here could not establish a role for RAGE in inflammation produced by intrapulmonary delivery of LPS. Together these data suggest that the contribution of RAGE to LPS responses *in vivo* depends on the body compartment and further indicate that the role of RAGE in antibacterial defense likely at least in part relies on the specific microorganism and the pathogen-associated molecular patterns they express.

Efferocytosis (the clearance of apoptotic cells via ingestion of apoptotic cells by macrophages and other phagocytic cells) is a major mechanism for the resolution of inflammation [46]. Of note, our laboratory demonstrated that during pneumonia, alveolar macophages are indispensable in the host response by means of their capacity to modulate inflammation via elimination of apoptotic neutrophils [47]. Previously, it was reported that RAGE participates in efferocytosis through binding to phosphatidylserine, the “eat me” signal highly expressed on apoptotic cells [48]. The lack of RAGE resulted in a decreased ability to engulf apoptotic cells *in vitro* and *in vivo* [46]. In our experiments, lung inflammation did not differ between the two mouse strains. In this context it is important to emphasize that the efferocytosis investigations were performed during sterile conditions. In our pneumonia model, the higher bacterial loads might have overruled this effect of RAGE deficiency, resulting in net similar lung inflammation and damage in the the lungs of RAGE^-/-^ mice compared with that of the wild-type mice.

The recruitment of neutrophils is an important part of host defense against pneumonia [49]. RAGE has been implicated in the regulation of cell migration. Indeed, RAGE^-/-^ mice had a lower number of adherent inflammatory cells on the peritoneum after CLP [20] and a reduction in neutrophil influx in the peritoneal cavity during thioglycollate peritonitis [19]. Furthermore, *in vivo* studies have suggested that RAGE is an endothelial counter receptor for the β2 integrin Mac-1 [19,50]. During pneumococcal pneumonia RAGE^-/-^ mice showed an attenuated influx of neutrophils into the lungs together with a decreased pneumococcal load [23]. Intratracheal delivered *E*. *coli* in mice resulted in lower neutrophil and MPO concentrations in BALF from RAGE^-/-^ mice [43]. Since CFUs were not reported in that study, it is not known whether the reduced neutrophil influx is at least in part due to a lower bacterial load. In contrast, we did not find an effect of RAGE deficiency on cell influx during *K*. *pneumoniae* pneumonia, as indicated by histopathology and pulmonary MPO concentrations. Moreover, leukocyte counts and differentials in BALF harvested after intrapulmonary delivery of *Klebsiella* LPS were similar in wild-type and RAGE^-/-^ mice which is in line with results of experiments with intratracheal administration of LPS from *E*. *coli* [43]. Together these data suggest that RAGE does not play a role of importance in leukocyte recruitment to the lungs during *K*. *pneumoniae* infection, and that the impact of RAGE on cell trafficking may depend on the inflammatory stimulus and the organ involved.

The current study is the first to establish that RAGE is important for antibacterial defense against *Klebsiella* pneumonia. We here show that RAGE plays a protective role during respiratory tract infection by a common Gram-negative causative pathogen, *K*. *pneumoniae*, by improving antibacterial defense in lungs and reducing bacterial dissemination. This could at least in part be explained by a better phagocytosis capacity of neutrophils in the presence of RAGE. Moreover, our results document that RAGE is not essential for the induction of excessive lung inflammation and injury.
